# Observational cohort study of the natural history of Niemann-Pick disease type C in the UK: a 5-year update from the UK clinical database

**DOI:** 10.1186/s12883-015-0511-1

**Published:** 2015-12-15

**Authors:** Jackie Imrie, Lesley Heptinstall, Stephen Knight, Kate Strong

**Affiliations:** NPUK, Vermont House, Concord, Washington, Tyne and Wear, NE37 2SQ UK; Department of Genetic Medicine, University of Manchester, Manchester, UK; The Care Forum, Bristol, UK

**Keywords:** Niemann-Pick disease type C, Natural history, *NPC1/NPC2*, Miglustat

## Abstract

**Background:**

Niemann-Pick disease type C (NP-C) is a rare neurovisceral lipid storage disorder characterised by progressive, disabling neurological symptoms and premature death in most patients. During the last decade, national cohort studies have accrued a great deal of data on the symptomatology and natural history of NP-C.

**Methods:**

In an observational cohort study, we present a substantial update based on the clinical presentation and follow-up of all *known* UK-based patients with a confirmed diagnosis of NP-C who have been tracked on an electronic database at the Department of Genetic Medicine, University of Manchester, UK. Patients were stratified according to accepted age-at-neurological-onset categories. Data on patients’ clinical signs and symptoms, medical history and genetic studies are summarised using descriptive methods.

**Results:**

A total of 146 patients with NP-C were included, representing the full known UK NP-C cohort, as observed from database information between 1999 and the end of 2011: 72 patients (49 %) were alive at the end of the observation period. Among a total of 116 patients (79 %) who possessed at least one identified, disease-causing NP-C gene mutation, 114 (98 %) had *NPC1* and two (2 %) had *NPC2* mutations. Overall, 53/194 (27 %) identified mutations were novel. Six patients (4 %) had an early, non-neurological neonatal onset form of NP-C. The numbers (%) of patients with accepted age-at-neurological onset forms were: 8 (5 %) early-infantile onset, 51 (35 %) late-infantile onset, 42 (29 %) juvenile onset, and 25 (17 %) adolescent/adult onset. Fourteen patients diagnosed based on visceral symptoms and/or sibling history, confirmed in most cases by genetic analysis, did not have any neurological manifestations at last follow up (11 patients with mean [SD] age at last follow up 2.5 [1.8] years: 3 with mean [SD] age at death 20.8 [15.9] years). A total of 51 patients (35 %) received miglustat therapy. The mean (SD) overall treatment duration up to the end of the observation period was 2.6 (2.3) years.

**Conclusions:**

This UK cohort is the largest national NP-C cohort reported to date, and confirms the wide phenotypic variability of the disease, as reported in other countries. Further analyses are required to assess the impact of miglustat therapy on neurological disease progression.

**Electronic supplementary material:**

The online version of this article (doi:10.1186/s12883-015-0511-1) contains supplementary material, which is available to authorized users.

## Background

Niemann-Pick disease type C (NP-C) is a rare neurovisceral lipid storage disorder characterised by progressive, disabling neurological symptoms and premature death in most patients [1–3]. It is caused by autosomal recessive inheritance of mutations in either of two genes (*NPC1* and *NPC2*), and has been estimated to affect one case in every 100,000–120,000 live births [1, 2, 4].

The clinical presentation of NP-C is highly heterogeneous, necessitating a multidisciplinary diagnostic process that takes into account clinical assessments, histological and electron microscopic tests, and biochemical and molecular genetic laboratory studies [1, 5]. Clinical work-up requires detection and recognition of numerous non-specific systemic and neurological signs and symptoms. While ancillary testing helps to narrow the differential diagnosis, final confirmation of NP-C requires demonstration of characteristic intralysosomal accumulation of unesterified cholesterol (based on filipin staining in cultured skin fibroblasts) and/or the identification of one or more disease-causing mutations in either the *NPC1* or *NPC2* genes [1, 2]. The demonstration of abnormal cholesterol homeostasis with impaired low-density lipoprotein (LDL)-induced cholesterol esterification can also provide supportive data in cases with an uncertain biochemical phenotype, but is rarely performed now [5, 6]. Newer screening and diagnostic tools have recently been developed. The NP-C suspicion index (SI) allows more rapid detection of patients who warrant further testing for NP-C [7–9]. Laboratory measurements of plasma oxysterols (particularly cholestane-3β,5α,6β-triol and 7-ketocholesterol) [10–12], and certain sphingolipids such as lyso-sphingosine [13, 14], have shown promise in allowing more rapid diagnosis in patients with suggestive clinical signs and symptoms. The increasing application of these newer measures is expected to increase the efficiency of diagnosis in NP-C.

Previous studies in cohorts of NP-C patients from France, Spain, Portugal and the USA have accrued data describing the biochemical and clinical phenotypes, genetics and natural history of NP-C [15–17]. NP-C has historically been considered a childhood-onset disease, but patients with late-onset symptoms are increasingly being detected due to the wider application of biochemical and genetic diagnostic techniques. In 2007, a retrospective case note review documented clinical signs and symptoms and subsequent disease course based on 94 NP-C patients diagnosed in the UK between 1999 and 2006 [15]. Data were available from approximately even numbers of patients with neonatal-onset (*n* = 33), childhood-onset (*n* = 31) and adolescent/adult-onset disease (*n* = 30), and a detection rate of 4–5 new cases per year between 1990 and 1999 was reported [15].

Major efforts have been made in the last decade to further consolidate clinical data from all known NP-C patients diagnosed and managed in the UK. This report provides an update of information from the UK NP-C database based on data collected between 1999 and the end of 2011, including a further 52 patients since the previous report in 2006 [15].

## Methods

### The UK NP-C database

We reviewed retrospective data for all UK-based patients with NP-C whose details were stored in a database maintained by the Niemann-Pick Disease Clinical Nurse Specialist at the Department of Genetic Medicine, University of Manchester, UK. All data were collected during clinical visits forming part of ongoing long-term care. The database contained information from all UK patients with a diagnosis of NP-C that had been confirmed using filipin staining, causal gene mutation analysis and/or esterification studies between 1999 and the end of 2011 (data cut-off). All patients were either referred or self-referred to the Nurse Specialist or Support Group.

### Diagnostic information

Laboratory diagnostic data for all patients are included from at least one of three laboratories in the UK and/or France that provide the required specialised testing. Diagnostic Laboratories in the UK and Lyon, France were involved independently in submitting information on cases they diagnosed to the database.

In general diagnostic information included findings from skin biopsy analyses (filipin staining and cholesterol esterification assays) from all UK patients with: 1) clinical symptoms suggestive of NP-C and; 2) raised plasma chitotriosidase (routinely checked alongside white-cell enzymes if laboratory analyses hinted at a possible lysosomal storage disease). *NPC1* gene sequencing analyses were conducted in all patients with positive filipin staining and/or cholesterol esterification findings, and some sibling cases. Patients in whom *NPC1* mutations were not identified, or on whom complementation studies have not been performed, underwent further investigations and *NPC2* gene sequencing.

### Clinical manifestations

During the observation period, information on key signs and symptoms of NP-C were recorded up to the last clinical follow up (i.e., last clinic visit and/or update of medical records in the database) in line with international guidelines for the diagnostic assessment and follow-up of the disease [1]. The following data types were included whenever possible: oculomotor signs (e.g. vertical supranuclear gaze palsy [VSGP]), neurological manifestations (e.g., cerebellar signs [ataxia, dysarthria, dysphagia, dyskinesia], seizures/cataplexy); spasticity; childhood developmental status (e.g. psychomotor delay and/or regression, learning disabilities); cognitive loss/problems, psychiatric abnormalities (e.g., psychosis, behavioural abnormalities); and systemic symptoms (e.g., hepatosplenomegaly, lung disease or neonatal cholestatic disease). Information on miglustat therapy was also included, where available, for all treated patients.

### Ethical data reporting

All information was accessed in accordance with applicable laws and ethical requirements for the study period concerned, and all study procedures, including informed consent for molecular genetic analyses, were conducted in line with ethical standards of the responsible institutional ethics committees and the Helsinki Declaration (1975) and subsequent revisions. All patients and/or their kin provided written informed consent for publication of individual clinical details, as presented in this report. Data reported previously for patients included in this cohort, based on publications by Lachmann et al. [18], Patterson et al. [19], and Patterson et al. [20], are identified where relevant.

### Data analysis

In recognition of international guidelines for the management of NP-C, patients are assessed based on neonatal presentation of NP-C (characterised mainly by systemic symptoms [splenomegaly, hepatomegaly, neonatal cholestatic disease and liver failure], and hereafter referred to as the ‘neonatal’ form), and as per accepted age subgroups based on onset of neurological manifestations (i.e., early infantile- [<2 years], late infantile- [2 to <6 years], juvenile- [6–15 years] and adolescent/adult-onset [>15 years]). Patients with a confirmed diagnosis but, as yet, no neurological symptoms, were also included in a ‘non neurological’ category.

All data analyses were exploratory in nature, and no statistical analyses of differences between patient subgroups were performed. Data are presented using descriptive statistics (mean, SD, median and range for continuous variables, and n (%) for categorical values). Patients for whom no numerical data values were available were treated as having ‘missing values’.

## Results

### Overall cohort characteristics

This UK cohort comprised a total of 146 NP-C patients born between 1954 and 2009, among whom 77 (53 %) were female and 69 (47 %) male. Patient demographics and general characteristics per patient subgroup are summarised in Table 1. Among a total of 112 patients (77 %) who possessed at least one identified, disease-causing NP-C gene mutation, 110 (98 %) had *NPC1* mutations and two (2 %) had *NPC2* mutations. In patients where genetic analyses did not reveal any known or identifiable novel mutations, diagnoses were based on filipin staining and ancillary methods combined with clinical examination findings and medical history.

**Table 1:** Patient demographics and general characteristics per age-at-onset subgroup

	Neonatal onset (*N* = 6)	Early infantile onset (*N* = 8)	Late-infantile onset (*N* = 51)	Juvenile onset (*N* = 42)	Adolescent/adult onset (*N* = 25)
Gender, n (%) female	3 (50)	7 (88)	28 (55)	25 (60)	10 (40)
Age, years:					
At neurological onset					
n^a^	–	8	50	40	18
Mean (SD)	–	1.1 (0.7)	4.1 (1.2)	9.4 (2.6)	24.2 (8.8)
At diagnosis					
n^a^	4^c^	7^c^	49	39	24
Mean (SD)	0.1 (0.1)	1.3 (1.5)	4.6 (5.5)	11.5 (7.4)	29.3 (9.3)
At last follow up^b^					
n^a^	–	–	19	21	17
Mean (SD)	–	–	11.6 (8.9)	20.2 (9.0)	39.5 (9.2)
At death					
n^a^	6	8	30	19	8
Mean (SD)	0.19 (0.22)	5.6 (2.0)	13.4 (6.7)	25.9 (8.9)	33.7 (6.2)
Number (%)^d^ with NP-C genetic information	1 (17)	8 (100)	41 (80)	36 (86)	16 (64)
Number (%)^d^ treated with miglustat	–	2 (25)	17 (33)	20 (48)	12 (48)

^a^Number of patients with available data; ^b^last follow up (data cut-off at end-2011) in living patients only; ^c^age at diagnosis not relevant for two neonatal patients and one early-infantile patient who were diagnosed post-mortem; ^d^percentages calculated relative to total number of patients per treatment subgroup

Six patients (4 %) had the visceral neonatal form of NP-C. The numbers (%) of patients per accepted age-at-neurological onset category were: early-infantile onset (*n* = 8 [5 %]), late-infantile onset (*n* = 51 [*n* = 35 %]), juvenile onset (*n* = 42 [29 %]); and adolescent/adult onset (*n* = 25 [17 %]). A total of 14 patients (10 %), most of whom were detected due to early visceral symptoms and three of whom had a sibling history of NP-C, had no neurological symptoms and are categorised herein as having non-neurological disease.

The overall mean (SD; range) ages at neurological onset and diagnosis were 8.8 (8.1; 0–40) years and 10.4 (11.5; 0–49.5) years, respectively. In general, age at diagnosis tended to increase in line with age at onset of neurological manifestations (Table 1): NP-C was diagnosed most quickly among patients with infantile onset, with the greatest delays to diagnosis recorded among patients with adolescent/adult onset. A total of 43 patients (29 %) were siblings who were also affected by NP-C. Based on all patients with available data, the mean time period between neurological disease onset and diagnosis was 1.73 (5.80) years, while this period among the sibling subgroup was 1.25 (4.75) years. In many cases, confirmation of a diagnosis of NP-C in one sibling led to a more rapid diagnosis in either younger or older siblings.

Overall, 72/146 (49 %) patients were alive at data cut-off. Among patients with available data, the mean age at last follow up (i.e., the latest clinical assessment before data cut-off at the end of 2011) ranged from 11.6 years in the late-infantile onset subgroup to 39.5 years in the adolescent/adult-onset subgroup. Mean ages at death among the age-at-onset subgroups ranged from 0.19 years among neonatal patients to 33.7 years in the adolescent/adult-onset subgroup. In non-neurological NP-C patients , the mean age at last follow-up among 11 living patients was 2.5 years (range 0.5–6.1). The mean age at death among three non-neurological patients who died was 20.8 (15.9) years (range 4.9–36.7).

### Neurological and psychiatric manifestations: overall cohort

The large majority of patients had at least one neurological manifestation commonly associated with NP-C at last follow up. A total of 19 patients (13 %) had no recorded manifestations, five of whom had the neonatal form of the disease.

**Fig. 1:**
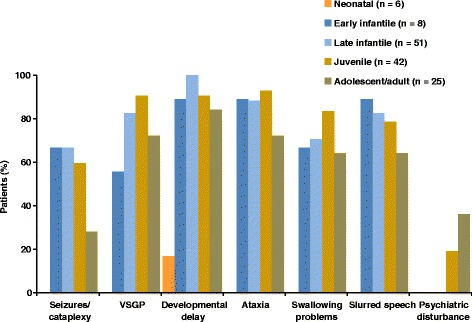
Occurrence of neurological and psychiatric manifestations per patient subgroup *Percentages calculated based on numbers of patients with available data*

Figure 1 summarises the prevalence of individual neurological symptoms per age at onset subgroup. Overall, developmental delay (during childhood) and/or cognitive deterioration were recorded in the greatest proportion of patients up to last follow up (in 119/146 patients [82 %]). In order of incidence, other common neurological signs were: ataxia (in 110 patients [76 %]), VSGP (103 patients [71 %]), dysarthria (99 patients [68 %]), dysphagia (93 patients [64 %]) and seizures/cataplexy (72 patients [50 %]).

Psychiatric disturbances were recorded in a total of 17 (12 %) patients. As could be expected, all cases in whom psychiatric disturbances were recorded were in the juvenile-onset or adolescent/adult-onset subgroups (incidence per subgroup 8/42 patients [19 %] and 9/25 patients [36 %], respectively).

### Visceral symptoms: overall cohort

**Fig. 2:**
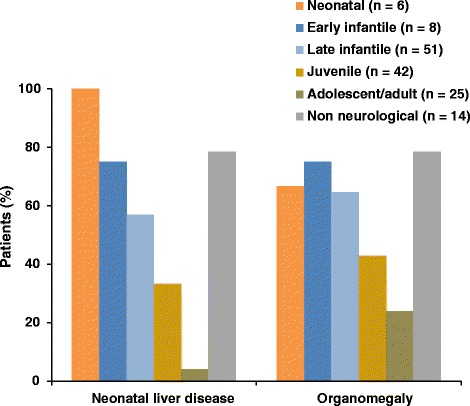
Occurrence of visceral symptoms per patient subgroup *Percentages calculated based on numbers of patients with available data*

Visceral symptoms of NP-C were recorded most frequently in patients with the neonatal visceral form of NP-C and in the early infantile-onset and non-neurological subgroups, and least frequently among adolescent/adult-onset patients (Fig. 2). Prolonged neonatal jaundice with or without neonatal liver disease was by far the most common visceral symptom, occurring in all neonatal onset, 6/8 (75 %) early-infantile onset, 29/51 (57 %) late-infantile onset, and 14/42 (33 %) juvenile-onset patients. Only one adolescent/adult-onset patient had a recorded history of neonatal jaundice. Organomegaly (hepatosplenomegaly in most cases) also tended to occur less and less frequently as age at neurological onset increased. All patients in whom no neurological manifestations have yet been recorded had a history of at least one visceral symptom, most commonly prolonged neonatal jaundice (in 5/14 [36 %] patients).

### Clinical symptomatology and outcomes in age-at-onset subgroups

#### Neonatal onset patients

All six patients with the neonatal form of NP-C had liver disease at or soon after birth (mean [SD] age 0.19 [0.22] years), with organomegaly also apparent in four cases and foetal ascites present in two (Table 2). Hepatosplenomegaly was also recorded in four cases. Patients with the severe neonatal-onset form of NP-C have been reported in other national cohorts to have a short lifespan [2, 16], and the same was true in this UK cohort. Two cases were stillborn and the other four died within 1–7 months of birth. Liver disease was the most frequently recorded cause of death (three patients), and one patient died due to failure to thrive and chest infection. One patient (patient 1) had a record of developmental delay from birth.

**Table 2:** Patients with neonatal NP-C

Patient number/Gender	Sibship	Date of birth	Age at diagnosis	Age at last FU/death^a^	Neonatal LD	HS/S	Seizures/cataplexy	VSGP	Dev. delay	Ataxia	Swallowing problems	Psychiatric disturbance	Slurred speech	Miglustat?	Age at 1^st^ miglustat start	Miglustat duration	Genetic mutations
1/M	–	2000	4 m	4 m^a^	PJ LD	HS	–	–	From birth	–	–	–	–	No	–	–	–
2/F	a	2001	PM	0^a^	FA	–	–	–	–	–	–	–	–	No	–	–	–
3/F	a	2002	PM	0^a^	FA	–	–	–	–	–	–	–	–	No	–	–	–
4/M	b	1985	Birth	1 m^a^	Yes	Yes	–	–	–	–	–	–	–	No	–	–	c.3501C > G(p.Phe1167Leu)/c.3501C > G(p.Phe1167Leu)
5/M	b	1985	Birth	2 m^a^	Yes	Yes	–	–	–	–	–	–	–	No	–	–	c.3501C > G(p.Phe1167Leu)/c.3501C > G(p.Phe1167Leu)
6/F	c	2009	2 m	7 m^a^	Yes	Yes	–	–	–	–	–	–	–	No	–	–	c.3020C > T(p.Pro1007Leu)/c.3020C > T(p.Pro1007Leu)

^a^Patient died; ‘–’, no data/not known; *C* cataplexy, *E* epilepsy, *FA* foetal ascites, *HS/S* hepatosplenomegaly/splenomegaly, *LD* liver disease, *m* months, *PJ* prolonged jaundice, *PM* post mortem, *y* years, *w* weeks

#### Early-infantile onset patients

All but one of the early-infantile onset patients were female (Table 3). The mean (SD; range) age at onset of neurological manifestations in this subgroup was 1.1 (0.7; 0–2.0) years. Overall, the mean (SD) time between onset of neurological manifestations and diagnosis was 0.26 (1.49) years, with diagnostic testing commenced based on recognition of visceral symptoms in four patients.

**Table 3:** Patients with early-infantile neurological onset

Patient number/Gender	Sibship	Date of birth	Age at diagnosis	Age at last FU (y, m)/death^b^	Neonatal LD	HS/S	Seizures/cataplexy	VSGP	Dev. delay	Ataxia	Swallowing problems	Psychiatric disturbance	Slurred speech	Miglustat?	Age at 1^st^ miglustat start	Miglustat duration	Genetic mutations
7/F	–	2000	4 m	3y 4 m^b^	PJ LD	Yes	C 2y 11 m	–	<18 m	<2y	13 m	–	<2y	No	–	–	c.3578_3591 + 9del/c.3578_3591 + 9del
8/F	–	2006	19 m	3y 9 m^b^	PJ	Yes	No	No	Yes	Yes	19 m	–	Yes	No	–	–	–
9/F	–	2005	3 m	4y 1 m^b^	PJ LD, LTx	Yes	–	–	Yes	Yes	Yes	–	Yes	No	–	–	c.1526A > C(p.Tyr509Ser/?
10/F	–	2003	6 m	4y 5 m^b^	PJ LD	Yes	E 3y 5 m	<18 m	Yes	Yes	3y	–	No speech	No	–	–	c.2801G > A(p.Arg934Gln)/c.2978del(p.Gly993Glu fsX4)
11/M	d	1990	PM	7y 8 m^b^	No	No	C 2y E 4y	Yes	<2y	<2y	5y	–	Yes	No	–	–	c.2819C > T(p.Ser940Leu/?^a^
12/F	e twin	1998	11 m	8y 5 m^b^	PJ	Yes	C 5y E 5y 10 m	3y	Yes	Never mobile	No	–	<3y	Yes	7y	1w	c.3107C > T(p.Thr1036Met/c.3557G > A(p.Arg 1186His)
13/F	e twin	1998	11 m	7y 1 m^b^	PJ	Yes	C 5y E 5y 10 m	3y	Yes	Never mobile	No	–	<3y	Yes	7y	1w	c.3107C > T(p.Thr1036Met/c.3557G > A(p.Arg 1186His)
14/F	–	1995	4y 5 m	6y 5 m^b^	No	No	C 4y E 5y	4y	1y 5 m	4y	5y	–	Yes	–	–	–	c.3503G > A(p.Cys1168Tyr)/c.3503G > A(p.Cys1168Tyr)

^a^Second mutant allele not found after full genome sequencing; ^b^Patient died; ‘–’, no data/not known; *C* cataplexy, *E* epilepsy, *FA* foetal ascites, *HS/S* hepatosplenomegaly/splenomegaly, *LD* liver disease, *LTx* liver transplant, *m* months, *PJ* prolonged jaundice, *PM* post mortem, *y* years, *w* weeks

The mean (SD) age at death was 5.6 (2.0) years. The most common recorded cause of death (7/8 cases [88 %]) was ‘NP-C’, reflecting a gradual and in some cases rapid deterioration, with eventual loss of all skills and bodily functions with no one apparent causative factor. However, the recorded causes of death could reflect the way death certificates are completed in the UK, where the primary disease is often stated as the causative factor.

A total of 6/8 patients (75 %) exhibited both prolonged neonatal jaundice and hepatosplenomegaly (Fig. 2). One patient (patient 9) underwent liver transplantation due to severe cholestatic liver disease.

Developmental delay, ataxia and dysarthria were the most commonly recorded neurological symptoms, each occurring in all eight patients (Fig. 1). Cataplexy/epileptic seizures and swallowing difficulties were each recorded in 6/8 (75 %) patients. Ophthalmic assessments revealed VSGP in 5/8 (63 %) patients.

#### Late-infantile onset patients

The mean (SD; range) age at neurological onset in this subgroup was 4.1 (1.2; 0.4–8.0) years, and the overall mean (SD) time period between neurological onset and diagnosis among patients with available data was 0.56 (5.25) years. Again, diagnostic testing was commenced in approximately half of this patient subgroup after initial recognition of visceral symptoms.

Overall, 30/51 (59 %) patients died before data cut-off at the end of 2011 (mean [SD] age at death 13.4 [6.7] years). ‘NP-C’ was listed as the cause of death in 26/30 patients (87 %) with available information. Pneumonia was listed specifically as the cause of death in two cases, but any association with dysphagia or previous food aspiration was not recorded.

Neurological manifestations were more common than visceral symptoms in this subgroup (Table 4). Again, developmental delay was recorded most frequently (in all 51 patients), followed by ataxia (in 45/51 [88 %]) and VSGP (in 42/51 [82 %]). Dysarthria, dysphagia and seizures/cataplexy were observed in 42/51 (82 %), 36/51 (71 %) and 34/51 (67 %) patients, respectively (Fig. 1).

**Table 4:** Patients with late-infantile neurological onset

Patient number/Gender	Sibship	Date of birth	Age at diagnosis	Age at last FU (y, m)/death^b^	Neonatal LD	HS/S	Seizures/cataplexy	VSGP	Dev. delay	Ataxia	Swallowing problems	Psychiatric disturbance	Slurred speech	Miglustat?	Age at 1^st^ miglustat start	Miglustat duration	Genetic mutations
15/M	f	2000	2y	5 y 4 m^b^	–	<1y 8 m	E <4y 11 m	–	<4y 6 m	<4y 6 m	<4y 6 m	–	<3y	No	–	–	c.3020C > T(p.Pro1007Leu)/c.3020C > T(p.Pro1007Leu)
16/F	–	2004	11 m	7y 2 m	PJ	S	No	5y	5y	4y	No	–	Mild <4y	Yes	6y	1y	–
17^a^/M	–	2005	1y 2 m	6y 7 m	HS	–	No	<6y	<5y	No	No	–	<6y	Yes	5y	1y	c.1526A > C(p.Tyr509Ser)/c.688_693del(p.Ser230_Val231del
18/F	–	2002	6 m	6y 4 m^b^	PJ	S	C 3y	Yes	3y	Yes	3y	–	3y	No	–	–	c.2008_2011del(p.Cys670ProfsX17/?^d^
19^a^/M	–	1990	15y	21y 2 m	–	–	E 15y	Yes	5y	Yes	–	–	–	Yes	16y	5y	c.2292G > A(p.Gly764Ala)/?
20/F	–	1987	6y	13y 6 m^b^	No	4y	–	7y	5y 5 m	7y	4y	–	Yes	No	–	–	c.2324A > C(p.Gln775Pro)/c.2956G > A(p.Gly986Ser)
21/M	g	2003	2y 11 m	7y^b^	PJ	HS	C 4y	2y 11 m	3y	<4y	Yes	–	<4y	No	–	–	c.2464_2465insT(p.Lys822IlefsX48)/c.2201G > T(p.ser734Ile)
22^a^/F	g	2005	4y	6y 10 m	Slight jaundice	No	No	<6y	<5y	<6y	No	–	<6y	Yes	3y	3y	c.2464_2465insT(p.Lys822IlefsX48)/c.2201G > T(p.ser734Ile)
23/F	d	1997	3y 9 m	12y 9 m^b^	No	No	C 6y	6y	6y 5 m	4y 7 m	7y	–	Yes	Yes	–	–	c.2819C > T(P.Ser940Leu)/?
24^a^/F	–	2001	7y	10y 10 m	No	No	E 9y C 8y	9y	<5y	9y	6y	–	<6y	Yes	8y	2y	c.3019C > g(p.Pro1007Ala)/?
25^a^/F	c/twin	2006	At birth	5y 6 m	PJ LD	HS	No	4y	<4y	<4y	No	–	4y	Yes	4y	1y	c.3020C > T(p.Pro1007Leu)/c.3020C > T(p.Pro1007Leu)
26^a^/F	c/twin	2006	At birth	5y 6 m	PJ LD	HS	No	4y	<4y	<4y	No	–	4y	Yes	4y	1y	c.3020C > T(p.Pro1007Leu)/c.3020C > T(p.Pro1007Leu)
27/F	f	2008	At birth	3y	Yes	Yes	No	No	<3y	Mild	No	–	No	Yes	0.5y	2.5y	c.3020C > T(p.Pro1007Leu)/c.3020C > T(p.Pro1007Leu)
28/M	–	1994	9 m	9y 3 m^b^	PJ LD	HS	C 4y E 7y	4y	1y	<5y	5y	–	5y	No	–	–	c.3182 T > C(p.Ile1061Thr)/?^d^
29/F	–	1990	9 m	9y 5 m^b^	PJ LD	No	Yes	<6y	<6y	6y 5 m	–	–	–	No	–	–	c.3182 T > C(p.Ile1061Thr)/?
30/F	–	1975	8y	28y^b^	PJ LD	HS	E 17y	8y	13y	5y	Yes	–	Yes	No	–	–	c.3182 T > C(p.Ile1061Thr)/?
31/F	–	2003	3 m	8y 6 m	PJ LD	At birth	E 8y	4y	<4y	<4y	<7y	–	5y	Yes	–	–	c.3182 T > C(p.Ile1061Thr)/?^d^
32//M	h	1989	2y	11y 2 m^b^	PJ LD	No	E 6y 5 m	Yes	4y	Yes	Yes	–	–	No	–	–	c.3182 T > C(p.Ile1061Thr)/?
33/M	h	1988	1y	8y 5 m^b^	PJ LD	No	C 6y E 6y	No	<5y	–	8y	–	–	No	–	–	c.3182 T > C(p.Ile1061Thr)/?
34/F	–	1990	4y 9 m	14y^b^	No	4y	C 7y E 8y	7y	6y	4y	8y	–	7y	No	–	–	c.3182 T > C(p.Ile1061Thr)/?
35/F	–	1989	7y	16y^b^	No	At birth	C 8y E 10y	5y	4y	4y 5 m	9y	–	Yes	No	–	–	c.3182 T > C(p.Ile1061Thr)/?
36/M	–	1987	8 m	12y 1 m^b^	PJ LD	At birth	C 5y 5 m E 7y 8 m	5y	5y	5y	9y	–	Yes	No	–	–	c.3182 T > C(p.Ile1061Thr)/?
37/M	–	1991	4y 3 m	9y^b^	PJ LD	HS 3y	C 5y	4y	4y 6 m	2y	5y	–	Yes	No	–	–	c.3182 T > C(p.Ile1061Thr)/?
38/M	–	–	16y	22y 11 m^b^	PJ	No	No	16y	8y	4y	17y	–	Yes	No	–	–	c.3182 T > C(p.Ile1061Thr)/?
39/M	–	2001	8 m	10y 6 m	PJ	HS	No	Yes	4y 6 m	Yes	No	–	No	Yes	7y	3y	c.3182 T > C(p.Ile1061Thr)/del10bp962
40/M	–	1995	10 m	10y^b^	PJ LD	S	C 4y 9 m	4y	>4y	Yes	Yes	–	Yes	No	–	–	c.3182 T > C(p.Ile1061Thr)/c.1142G > A(p.Trp381X)
41/F	–	1996	12 m	11y 9 m^b^	No	HS 9 m	C 5y 6 m	5y	5y	5y 6 m	No	–	11y	Yes	7y	4y	c.3182 T > C(p.Ile1061Thr)/c.2656G > C(p.Gly886Arg)
42/F	–	1983	9y 5 m	25y 7 m^b^	No	S 9y 6 m	E 5y	Yes	Yes	12y	Yes	–	Yes	No	–	–	c.3182 T > C(p.Ile1061Thr)/c.3019C > G(p.Pro1007Ala)
43/F	–	1993	6y	9y 6 m^b^	PJ	5y	C 5y E6y	5y	5y	5y	6y	–	Yes	No	–	–	c.3182 T > C(p.Ile1061Thr)/c.3107C > T(p.Thr1036Met)
44^a^/M	–	2007	4 m	4y 1 m	NC	Yes	No	No	<3y	<3y	No	–	<3y	Yes	2y	2y	c.3182 T > C(p.Ile1061Thr)/c.3107C > T(p.Thr1036Met)
45^a^/M	–	1998	4 m	13y 9 m	PJ	Yes	C 8y	5y	9y	No	No	–	No	Yes	5y	8y	c.3182 T > C(p.Ile1061Thr)/c.3175C > T(p.Arg1059X)
46/F	–	1996	7y 8 m	15y 6 m^b^	No	No	C 8y	7y 8 m	7y 8 m	5y	8y 5 m	–	Yes	No	–	–	c.3182 T > C(p.Ile1061Thr)/c.3182 T > C(p.Ile1061Thr)
47^a^/M	–	2006	At birth	5y	PJ LD	Yes	C <4y	No	3y	Yes	<5y	–	<4y	Yes	4y	1y	c.3182 T > C(p.Ile1061Thr)/c.3422 T > G(p.Val1141Gly)
48/F	–	1982	3y	29y 4 m	PJ	HS at birth	No	3y	3y	5y	18y	–	Yes	Yes	–	–	c.3182 T > C(p.Ile1061Thr)/c.3467A > G(p.Asn1156Ser)
49^a^/F	–	2001	12 m	10y 7 m^b^	No	S	C 9y	4y	4y	4y	7y	–	6y	No	–	–	c.3182 T > C(p.Ile1061Thr)/c.3591 + 4delA
50^a^/M	–	2004	1y 11 m	7y 5 m^b^	No	6 m	C 4y	4y	3y 5 m	3y	4y 5 m	–	Yes	Yes	5y	2y	c.3259 T > C(p.Phe1087Leu)/c.2516 T > G(p.Ile839Arg)
51/F	i	1984	8y 6 m	27y 5 m	No	2y	C 5y	8y	13y	5y	13y	–	11y	No	–	–	c.3467A > G(p.Asn1156Ser)/?^d^
52/F	i	1978	14y 5 m	33y 5 m^b^	No	No	E 13y	14y	5y	13y	13y	–	12y	No	–	–	c.3467A > G(p.Asn1156Ser)/?^d^
53/F	j	1995	8y 4 m	16y 11 m	No	No	E 7y 5 m C 8y	6y 9 m	3y 5 m	4y	8y 6 m	–	Yes	No	–	–	c.3591 + 4delA/?^d^
54^a^/M	–	2006	At birth	5y 5 m	PJ LD	Yes	No	No	Yes	No	Yes	–	Yes	No	–	–	c.58G > T(p.Glu20X)/c.58G > T(p.Glu20X) [NPC2]
55/M	k	2000	–	10y 5 m^b^	No	No	C 6y	5y	5y	5y	5y	–	Yes	No	–	–	–
56/M	–	2005	3y 10 m	–	Yes	–	–	–	4y	–	–	–	4y	No	–	–	–
57/F	l	–	2y	6y^b^	No	HS 6 m	No	3y	2y 5 m	2y 5 m	5y	–	No	No	–	–	–
58/M	–	–	9y 5 m	15y^b^	No	No	C + E 5y	6y	6y	4y	10y	–	Yes	No	–	–	–
59/F	–	1987	8y	17y 6 m^b^	No	4y	C 5y E 10y	7y	5y	6y	11y	–	Yes	No	–	–	–
60/M	b	1983	8y	16y^b^	PJ	No	E 16y	Yes	8y	5y	12y	–	Yes	No	–	–	c.3501C > G(p.Phe1167Leu)/c.3501C > G(p.Phe1167Leu)
61/M	l	1990	7y 1 m	17y 5 m^b^	No	S 2y 6 m	C 10y	7y 1 m	4y 5 m	5y 5 m	10y	–	Yes	No	–	–	–
62/F	–	1993	5y	9y 5 m^b^	PJ	HS 5y 5 m	C 5y	No	18 m	4y	No	–	Yes	No	–	–	–
63/M	k	1998	–	–	–	–	–	Yes	Yes	Yes	Yes	–	Yes	No	–	–	–
64/F	–	1982	29y	29y 6 m	No	No	No	29y	Yes	Yes	20y	–	24y	No	–	–	c.3022A > C(p.Asn108His)/c.182 T > C(p.Ile 1061Thr)
65/F	–	2008	2y	3y 9 m	Yes	HS	No	No	Yes	No	No	–	No	No	–	–	[*NPC2*]^c^

^a^Patients included in previously reported NPC Registry baseline characteristics study [19]; ^b^patient died; ^c^mutation found but information not accessible during observation period; ^d^second mutant allele not found after full genome sequencing. ‘–’, no data/not known; *C* cataplexy, *E* epilepsy, *FA* foetal ascites, *HM* hepatomegaly, *HS/S* hepatosplenomegaly/splenomegaly, *LD* liver disease, *LTx* liver transplant, *m* months, *NC* neonatal cholestasis, *PJ* prolonged jaundice, *PM* post mortem, *y* years, *w* weeks

The only two patients in the UK cohort who had *NPC2* mutations were in this age-at-onset subgroup (patients 54 and 65), both of whom displayed neonatal jaundice and hepatosplenomegaly, as was common among other late-infantile onset patients. However, both of these patients displayed relatively few typical neurological manifestations: patient 54 had developmental delay, dysarthria and dysphagia, and only developmental delay was recorded in patient 65.

Overall, hepatosplenomegaly was the most common visceral symptom in this patient subgroup, recorded in 32/51 (63 %) patients. A history of neonatal jaundice was recorded in 29/51 (57 %) (Fig. 2).

#### Juvenile-onset patients

Among juvenile-onset patients, neurological manifestations were first noted at a mean (SD; range) age of 9.4 (2.6; 5.0–15.0) years. The mean (SD) time between neurological onset and diagnosis was 1.64 (6.09) years. In this subgroup, diagnostic testing was commenced based on the appearance of neurological signs in the majority (approximately three-quarters) of cases.

In total, 19/42 (45 %) patients had died by data cut-off (mean [SD] age at death, 25.9 [8.9] years) (Table 5). As for late-infantile onset patients, most deaths (11/18 evaluable patients [61 %]) in this subgroup were recorded as being due to ‘NP-C’. Four of the 18 (22 %) patients with available information died due to respiratory-related complications but again, no associations with dysphagia or previous aspiration were noted.

**Table 5:** Patients with juvenile neurological onset

Patient number/Gender	Sibship	Date of birth	Age at diagnosis	Age at last FU (y, m)/death^c^	Neonatal LD	HS/S	Seizures/cataplexy	VSGP	Dev. delay/cognitive problems	Ataxia	Swallowing problems	Psychiatric disturbance	Slurred speech	Miglustat?	Age at 1^st^ miglustat start	Miglustat duration	Genetic mutations
66/F	–	1970	24y 5 m	41y 2 m	No	Yes	C 24y	12y	11y	24y	24y	32y	24y	Yes	32y	–	c.1211G > A(p.Arg404Gln)/?^d^
67/F	–	1995	10y 1 m	16y 3 m	No	No	E 10y 3 m	10y 1 m	8y	10y	13y	No	Yes	Yes	12y	4y	c.1552C > T(p.Arg518Trp)/c.283 T > C(p.Ser95Pro)
68/F	–	1996	10y 6 m	15y 1 m	–	–	<10y	Yes	<10y	<10y	<10y	No	<10y	No	–	–	c.2848G > A(p.Val950Met)/?
69/F	–	1994	14y	17y 10 m	No	No	No	Yes	10y	Yes	Yes	–	6y	No	–	–	c.2974G > T(p.Gly992Trp)/?
70/F	–	1983	14y	21y 11 m^c^	No	No	E 12y	Yes	8y	11y	17y	No	Yes	No	–	–	c.3019C > G(p.Pro1007Ala)/?
71^a^/M	–	1991	17y	20y 4 m	–	–	E 12y	Yes	At birth	Yes	Yes	Yes	Yes	Yes	18y	2y	c.3019C > G(p.Pro1007Ala)/c.1553G > A(p.Arg518Gln
72/F	–	1986	2 m	25y 10 m	FA LD	At birth	E 14y	11y	11y	14y	15y	No	<14y	No	–	–	c.3176G > A(p.Arg1059Gln)/?
73/M	–	1996	14y	15y 5 m	No	No	No	12y	7y	7y	13y	No	12y	Yes	14y	1y	c.3182 T > C(p.Ile1061Thr)/?
74/M	–	–	7y 5 m	20y^c^	PJ	HS	No	7y	6y 5 m	7y	8y	No	Yes	No	–	–	c.3182 T > C(p.Ile1061Thr)/?
75/F	–	1986	10y	19y 2 m^c^	No	No	C 11y	10y	7y	7y	11y	No	Yes	No	–	–	c.3182 T > C(p.Ile1061Thr)/?
76/M	–	2000	7y	11y 1 m^c^	–	–	E 7y	Yes	Yes	Yes	Yes	No	Yes	No	–	–	c.3182 T > C(p.Ile1061Thr)/?
77^a^/F	–	1996	4y 5 m	15y 4 m	PJ	HS 4y 5 m	E 12y	7y	7y	6y	No	No	No	Yes	7y	8y	c.3182 T > C(p.Ile1061Thr)/c.2801G > A(p.Arg934Gln)
78^a^/F	–	2002	2 m	9y 10 m	PJ	HS	C 6y	Yes	6y	No	No	No	No	Yes	7y	2y	c.3182 T > C(p.Ile1061Thr)/c.2819C > T(p.Ser940Leu)
79/F	–	1985	4y 1 m	26y 4 m	PJ LD	HS 11y	C 7y E 17y	11y	13y	13y	16y	No	No	Yes	–	–	c.3182 T > C(p.Ile1061Thr)/c.2974G > T(p.Gly992Trp)
80/M	m	1978	12y	18y 8 m^c^	No	S 2–10y	E 11y	10y	8y	10y	18y	No	Yes	No	–	–	c.3182 T > C(p.Ile1061Thr)/c.3019C > G(p.Pro1007Ala
81/M	–	2003	6 m	8y 8 m	PJ LD	HS	–	–	No	–	–	–	–	Yes	7y	1y	c.3182 T > C(p.Ile1061Thr)/ c.3182 T > C(p.Ile1061Thr)
82/M	–	1970	At birth	32y^c^	No	No	E 22y	Yes	12y	Yes	27y	No	Yes	No	–	–	c.3182 T > C(p.Ile1061Thr)/ c.3182 T > C(p.Ile1061Thr)
83/F	–	1991	13y	18y 2 m^c^	No	No	No	Yes	10y	14y	14y 5 m	No	Yes	No	–	–	c.3182 T > C(p.Ile1061Thr)/ c.3182 T > C(p.Ile1061Thr)
84^b^/M	–	1988	12y	23y 7 m	No	S 7 y	No	11y 9 m	17y	17y	No	No	No	Yes	21y	2y	c.3182 T > C(p.Ile1061Thr)/ c.3182 T > C(p.Ile1061Thr)
85^b^/F	–	1991	6y 5 m	20y 1 m	No	S 5y 5 m	No	8y 3 m	11y 1 m	9y	19y	No	No	Yes	12y	8y	c.3182 T > C(p.Ile1061Thr)/c.3493G > A(p.Val1165Met)
86/F	–	2005	8w	6y 6 m	PJ LD	HS	No	Mild 6y	No	No	No	No	No	Yes	4y	2y	c.3182 T > C(p.Ile1061Thr)/c.350-351delAG
87/F	–	1986	17y	25y 5 m	No	No	E 16y	Yes	5y (FAS)	<16y	<16y	–	Yes	No	–	–	c.3182 T > C(p.Ile1061Thr)/c.3566A > G(p.Glu1189Gly)
88/M	–	1985	18y	26y 7 m	No	S 18 y	No	15y	13y	13y	18y	18y 6 m	18y	Yes	–	–	c.3182 T > C(p.Ile1061Thr)/c.3566A > G(p.Glu1189Gly)
89^a^/F	–	1994	10y 4 m	17y 9 m	No	No	C <4y E <17y	<17y	<10y 4 m	<10y 4 m	<17y	No	<17y	Yes	14y	3y	c.3182 T > C(p.Ile1061Thr)/c.410C > T(p.Thr137Met)
90/F	–	1983	15y	22y 8 m^c^	PJ	Birth	E 14y	11y	8y	11y	16y	No	Yes	No	–	–	c.3263A > G(p.Tyr1088Cys)/c.1201C > A(p.Pro401Thr)
91/M	–	1989	17y	23y	No	No	No	Yes	8y	9y	18y	17y	17y	Yes	18y	3 m	c.3493A > G(p.Val1165Met)/c.3493A > G(p.Val1165Met)
92/F	b	1990	3 m	17y 2 m^c^	PJ	No	E 15y	11y	10y	10y 5 m	15y	No	Yes	Yes	–	–	c.3501C > G(p.Phe1167Leu)/c.3501C > G(p.Phe1167Leu)
93/F	j	1998	10y	13y 6 m	No	No	Yes	Yes	Yes	Yes	Yes	No	Yes	Yes	10y	3y	c.3591 + 4delAla/?
94/F	–	1997	10y	14y 8 m^c^	No	No	C + E 10y	No	<10y	<10y	Yes Gx	No	Yes	No	–	–	c.3019C > G(p.Pro1007Ala)/c.2464-2465insT
95/M	–	1998	12y	13y 6 m	No	No	C 11y	11y	9y	Tremor 11y	No	No	<5y	Yes	13y	6 m	–
96/F	–	1996	9y	15y 8 m^c^	Mild jaundice	S	–	Yes	Yes	Yes	Yes Gx	No	9y	No	–	–	–
97/F	–	1991	16y	17y 6 m^c^	–	–	S, C	Yes	<15y	<15y	<17y	No	Yes	No	–	–	–
98/M	–	1967	PM	25y^c^	PJ	S 2 y	No	Yes	11y	2y	14y	25y	Yes	No	–	–	–
99/F	–	1972	16y	39y 11 m	PJ	No	No	16y	11y	28y	30y	30y	Yes	Yes	–	–	c.1211G > A(p.Arg404Gln)/c.1133 T > C(P.Val378Ala)
100/M	–	1971	14y	29y 2 m^c^	No	13y 5 m	13y	13y 5 m	13y	13y 5 m	13y	No	Yes	No	–	–	–
101/F	–	1963	18y	40y 3 m^c^	PJ	No	E 31y	16y	12y	16y	23y	16y	Yes	No	–	–	c.1843C > T(p.Arg615Cys)/c.2972-2973del(p.Gln991ArgfsX15)
102/F	n	–	Teens	23y^c^	No	No	–	No	Teens	Teens	Teens	23y	–	No	–	–	c.3182 T > C(p.Ile1061Thr)/?
103/M	–	1987	20y	24y 5 m	No	No	No	<19y	No	13y	22y	No	13y	Yes	22y	2y	c.2861C > T(p.Ser954Leu)/c.3107C > T(p.Thr1036Met)
104/M	–	1972	6y	39y 8 m	No	HS 5y	No	No	No	6y	No	No	No	No	–	–	c.1844G > T(p.Arg615Leu)/c.1844G > T(p.Arg615Leu)
105/M	–	1969	Early 20s	37y 8 m^c^	PJ	S 4y	E 30y	Yes	11y	20s	Yes	No	Yes	No	–	–	–
106^a^/F	o	1976	27y	35y 10 m	No	No	No	27y	13y	26y	26y	No	27y	Yes	–	–	c.1552C > T(p.Arg518Trp)/c.1552C > T(p.Arg518Trp)
107/M	o	1972	30y	37y 2 m^c^	No	No	Teens	26y	26y	26y	27y	25y	26y	No	–	–	c.1552C > T(p.Arg518Trp)/c.1552C > T(p.Arg518Trp)

^a^Patients included in previously reported NPC Registry baseline characteristics study [19]; ^b^Patients included in previously reported NPC Registry baseline and longitudinal data study [19, 20]; ^c^patient died; ^d^second mutant allele not found after full genome sequencing. ‘–’, no data/not known; *C* cataplexy, *E* epilepsy, *FA* foetal ascites, *Gx* gastrostomy, *HM* hepatomegaly, *HS/S* hepatosplenomegaly/splenomegaly, *LD* liver disease, *LTx* liver transplant, *m* months, *NC* neonatal cholestasis, *PJ* prolonged jaundice, *PM* post mortem, *y* years, *w* weeks

Neurological manifestations were substantially more common than visceral symptoms in this subgroup. Ataxia was the most common neurological manifestation (in 39/42 [93 %] patients), followed by VSGP and childhood developmental delay or cognitive problems (both in 38/42 [91 %]), dysphagia (35/42 [83 %]), dysarthria (33/42 [79 %]) and seizures/cataplexy (25/42 [60 %]) (Fig. 1). Unlike younger patient subgroups, psychiatric disturbances were recorded in juvenile-onset patients (8/42 [19 %] cases). The age at onset of psychiatric disturbances ranged from 16 to 32 years.

Neonatal jaundice and/or cholestatic liver disease were recorded in 14/42 (33 %) patients, and organomegaly was seen in 18/42 (43 %) patients (Fig. 2). While no specific neurological signs were recorded in one patient (patient 81), evidence of neurological involvement was documented at the local treatment centre. However, it is not possible to report this patient’s presenting neurological manifestation as he was lost to follow up.

#### Adolescent/adult-onset patients

Overall, patients with neurological onset during adolescence or adulthood were characterized by insidious onset and slow disease progression (Table 6). Neurological onset in this older age subgroup occurred at a mean (SD; range) age of 24.2 (8.8; 15.0–40.0) years. The mean (SD) period between neurological onset and diagnosis was 6.0 (6.26) years, and all evaluable patients in this subgroup were diagnosed after the appearance of neurological symptoms. By data cut-off, a total of 8/24 (32 %) patients had died (mean [SD] age at death, 33.7 [6.2] years). Among patients with available records, seven were recorded as being due to NP-C.

**Table 6:** Patients with adolescent-adult neurological onset

Patient number/Gender	Sibship	Date of birth	Age at diagnosis	Age at last FU (y, m)/death^c^	Neonatal LD	HS/S	Seizures/cataplexy	VSGP	Dev. delay/cognitive problems	Ataxia	Swallowing problems	Psychiatric disturbance	Slurred speech	Miglustat?	Age at 1^st^ miglustat start	Miglustat duration	Genetic mutations
108^b^/M	m	1976	23y	39y	PJ	No	No	17y	17y	25y	25y	No	Yes	Yes	32y	3y	c.3182 T > C(p.Ile1061Thr)/c.3019C > G(p.Pro1007Ala)
109/F	–	1975	18y	30y^c^	No	No	E 18y 6 m	18y	15y	15y	24y	18y	Yes	No	–	–	c.3182 T > C(p.Ile1061Thr)/c.3019C > G(p.Pro1007Ala)
11/M	–	1978	20y	27y 3 m^c^	No	No	No	<24y	<20y	No	23y	Yes	–	No	–	–	–
111/F	–	1975	25y 6 m	30y^c^	No	No	C 25y	25y	16y	24y	23y	No	23y	No	–	–	–
112/M	n	1971	24y	40y 2 m	No	No	No	16y	24y	<24y	No	29y	32y	No	–	–	c.3182 T > C(p.Ile1061Thr)/?
113/M	p	1968	19y	43y 2 m^c^	No	S 18y	E 17y	25y	18y	18y	25y	17y	18y	No	–	–	c.1843C > T(p.Arg615Cys)/c.3289-3291del(p.Asp1097del)
114^a^/M	–	1987	22y	24y 11 m	No	No	No	22y	15y	15y	23y	No	18y	Yes	24y	0.5y	–
115/M	p	1969	18y	28y 8 m^c^	No	S 1y 4 m	No	Yes	Yes	Yes	25y	28y	Yes	No	–	–	c.1843C > T(p.Arg615Cys)/c.3289-3291del(p.Asp1097del)
116/F	–	1963	38y	48y 6 m	No	No	No	<34y	37y	34y	39y	No	34y	Yes	39y^e^	–	c.1133 T > C(p.Val378Ala)/c.422_423dup(p.Lys142X)
117^a^/F	o	1978	25y	33y 11 m	No	No	No	No	25y	25y	32y	No	32y	Yes	29y	4y	c.1552C > T(p.Arg518Trp)/c.1552C > T(p.Arg518Trp)
118/M	–	–	27y	35y 4 m	–	Yes	Teens	No	Yes	Yes	Yes	20s	Yes	No	–	–	–
119^d^/F	–	1966	32y	42y	No	No	No	Yes	<18y	29y	38y	No	38y	Yes	37y	8	c.3182 T > C(p.Ile1061Thr)/?
120/M	–	1972	29y	39y 10 m^c^	–	–	–	–	Yes	–	–	–	–	No	–	–	–
121^a^/M	–	1982	20y	29y 2 m	No	No	No	–	Yes	–	–	–	Yes	No	–	–	–
122/M	–	1964	31y	48y 6 m	No	Yes	No	Yes	46y	No	Yes	No	No	Yes	42y	5y	–
123^a^/M	q	1967	40y	44y 5 m	–	No	Tremor	<40y	<40y	<40y	No	No	No	Yes	43y	1y	c.1408G > C(p .Ala 470 Pro/c.1816G > C (p.Glu608Gln)
124/F	–	1961	49y	50y 11 m	No	No	No	46y	42y	42y	49y	40y	42y	Yes	49y	1y	c.2000C > G(p.Ser667Trp)/?
125/F	r	1981	29y	30y 3 m^c^	No	20y	No	20s	20s	20s	20s	Teens	20s	–	–	–	c.2764C > T(p.Gln922X)/c.1133 T > C(p.Val378Ala)
126/M	r	1985	25y	26y 8 m	No	No	No	25y	Mild 25y	No	No	No	No	Yes	25y	1y	c.2764C > T(p.Gln922X)/c.1133 T > C(p.Val378Ala)
127/M	–	1969	42y	42y 6 m	–	No	Tremor (20s)	<34y	<34y	Yes	40y	No	Yes	No	43y	–	c.2903A > G(p.Asn968Ser)/c.3182 T > C(p.Ile1061Thr)
128/F	–	1954	49y 6 m	57y 6 m	No	No	No	Yes	Yes	20s	No	No	20s	No	–	–	c.3182 T > C(p.Ile1061Thr)/?
129/F	–	–	PM	40y^c^	–	–	–	–	–	–	–	–	–	–	–	–	c.3182 T > C(p.Ile1061Thr)/c.2861C > T(p.Ser954Leu)
130/M	q	–	40y	41y 9 m	–	40y	Tremor (35y)	Yes	No	Yes	40y	No	No	Yes	39y	–	c.1408G > C(p .Ala 470 Pro/c.1816G > C (p.Glu608Gln)
131/F	–	–	27y	27y 8 m	No	No	No	No	No	25y	No	27y	No	No	–	–	c.3022A > C p (Asn 1008 His)/c.3182 T > C p.(Ile 1061Thr)
132^a^/M	s	1974	Early 30s	–	–	–	–	–	20y	–	–	–	–	Yes	–	–	c.2336del (p.Phe779SerfsX2)/c.2621A > T(p.Asp874Val)

^a^Patients included in previously reported NPC Registry baseline characteristics study [11]; ^b^Patients included in previously reported NPC Registry baseline and longitudinal data study [11, 12]; ^c^patient died; ^d^patient 119 F previously subject of case report by Lachmann et al. [10]; ^e^treatment interrupted (first treatment start at 39 y, second treatment start at 46 y); ‘–’, no data/not known; *C* cataplexy, *E* epilepsy, *FA* foetal ascites, *HM* hepatomegaly, *HS/S* hepatosplenomegaly/splenomegaly, *LD* liver disease, *LTx* liver transplant, *m* months, *NC* neonatal cholestasis, *PJ* prolonged jaundice, *PM* post mortem, *y* years, *w* weeks

A history of developmental delay and/or cognitive deterioration was the most frequently recorded neurological manifestation (in 21/25 [84 %] patients), followed by VSGP and ataxia (both in 18/25 [72 %] patients). Dysarthria, dysphagia and seizures/cataplexy were present in 28–64 % of patients. Among those with available information on time of onset of specific manifestations, the majority of neurological signs seemed to appear during adulthood. In particular, patient 124 had no observable neurological symptoms up until her 4^th^ decade of life, during which a full spectrum of characteristic neurological signs as well as psychiatric problems occurred. Patient 129 had a long history of neurological problems but no data on age at onset or recorded details on specific neurological manifestations. Patient 132 displayed cognitive deterioration since his twenties, but again, no information is available regarding specific neurological manifestations.

As in the juvenile-onset subgroup, psychiatric disturbances and/or cognitive deterioration were prominent in the adolescent/adult-onset subgroups, recorded in a total of nine patients (36 %). The age at onset of psychiatric disturbances ranged between 17 and 40 years.

There was only one historical record of neonatal jaundice in this subgroup, and organomegaly was recorded in a total of 6/25 (24 %) patients. Splenomegaly or hepatosplenomegaly were observed in three patients during adulthood (at age 18–40 years; patients 113, 125 and 130), and splenomegaly was recorded during the neonatal period in one patient (patient 115 at age 16 months).

#### Patients with no neurological manifestations

There were a total of 14 patients who had not displayed any neurological manifestations by data cut-off (Table 7). Genetic analyses have revealed *NPC1* mutations in the majority (*n* = 10; 71 %) of these patients, confirming NP-C. Diagnoses among those without identified gene mutations were confirmed based on filipin staining. All of these patients had at least one early visceral symptom of NP-C: neonatal liver disease (mostly prolonged jaundice) and organomegaly both occurred in 11/14 (79 %) patients in this subgroup. Two patients (141 and 143) died in their 2^nd^ and 3^rd^ decades of life due to non NP-C related causes, and patient 146 died aged 4 years due to liver cancer. The remainder of non-neurological patients were alive at data cut-off. The mean (SD) age at last follow up prior to data cut off was 2.5 (1.8) years (range 0.5–6.1 years; *n* = 11). Among the three patients who died, mean age at death was 20.8 (15.9) years (range 4.9–36.7).

**Table 7:** Patients with no neurological symptoms

Patient number/Gender	Sibship	Date of birth	Age at diagnosis	Age at last FU (y, m)/death^a^	Neonatal LD	HS/S	Seizures/cataplexy	VSGP	Dev. delay/cognitive problems	Ataxia	Swallowing problems	Psychiatric disturbance	Slurred speech	Genetic mutations
133/M	–	2010	4 m	6 m	Yes	No	No	No	No	No	No	No	–	–
134/M	–	2010	8 m	8 m	Yes	–	–	–	–	–	–	–	–	c.3182 T > C(p.Ile1061Thr)/?
135/M	–	2010	1y	1y	Yes (LTx)	Yes	No	No	No	No	No	No	No	–
136/M	–	2006	6 m	2y 5 m	PJ LD	<4y	No	No	No	No	No	No	No	c.3182 T > C (p.Ile1061Thr)/c.3182 T > C (p.Ile1061Thr)
137/M	–	2003	2y6m	6y 1 m	No	Yes	No	No	No	No	No	No	No	c.3182 T > C (p.Ile1061Thr)/c.3467A > G (p.Asn1156Ser)
138/M	–	2009	4 m	2y 1 m	PJ LD	Yes	No	No	No	No	No	No	No	c.3182 T > C (p.Ile1061Thr)/?
139/M	t	2010	<1y	1y	Yes	Yes	No	No	No	No	No	No	No	c.3182 T > C (p.Ile1061Thr)/c.3289G > A (p.Asp1097Asn)
140/M	t	2006	–	5y	No	No	No	No	No	No	No	No	No	c.3182 T > C (p.Ile1061Thr)/c.3289G > A (p.Asp1097Asn)
141/F	s	1971	1y5m	36y 8 m^a^	PJ LD	Birth	No	No	Yes	No	No	No	No	c.2336del (p.Phe779SerfsX2)/c.2621A > T (p.Asp874Val)
142/F	–	2008	2y5m	3y	Yes	Yes	No	No	No	No	No	No	No	c.3182 T > C (p.Ile1061Thr)/c.3259 T > C (p.Phe1087Leu)
143/M	–	1987	16y	20y 10 m^a^	PJ	Birth	No	No	No	No	No	No	No	c.2621A > T (p.Asp874Val)/c.3591 + 4 delA
144/M	–	2007	1y9m	4y	No	Yes	No	No	No	No	No	No	No	–
145/M	–	2009	4 m	2y	Yes	Yes	No	No	No	No	No	No	No	–
146/F	–	2004	7 m	4y 11 m^a^	PJ	Yes	No	No	No	No	No	–	No	c.3083-3084delP.Gly1028AlafsX22)/c.2201G > T(p.Ser734Ile)

^a^Patient died; ‘–’, no data/not known; *HS/S* hepatosplenomegaly/splenomegaly, *LD* liver disease, *LTx* liver transplant, *m* months, *PJ* prolonged jaundice, *y* years, *w* weeks. Note that none of the non-neurological patients had received miglustat by data cut-off end-2011

### Genetics

Overall, 116/146 (79 %) patients had at least one identified NP-C gene mutation (Tables 2–6**)**. Two mutations were recorded in 78/146 (53 %) patients and one mutation was recorded in 38/146 (26 %) patients. Most patients (98 %) with recorded mutant alleles had *NPC1* mutations, and two patients (2 %) had *NPC2* mutations (one with homozygous c.58G > T(p.Glu20X) mutant alleles and one with a single identified mutation for which information was not available). Thirty patients (21 %) had no recorded information regarding identified NP-C gene mutations. This number may be reduced in future with the increasing availability of next-generation sequencing methods.

Amongst a total of 194 identified mutant NP-C gene alleles, 53 have been classified as novel mutations. The common I1061Thr mutation was recorded in a total of 55 (38 %) patients in the whole cohort (heterozygous in 89 % and homozygous in 11 % of cases). Among the age-at-neurological onset subgroups, I1061Thr mutant alleles were mainly observed in patients with the late infantile- (23/51 [45 %]) and juvenile-onset forms (18/42 [43 %]). A lower proportion of patients in the adolescent-adult onset group had this mutation (7/25 [28 %]). However, the prevalence of I1061Thr mutations was particularly high among non-neurological patients (*n* = 7 [50 %]; six heterozygotes and one homozygote). One patient with a I1061Thr allele (patient 73), also had a c.882-40 T > A mutant allele, and family studies detected this genotype in the child’s father. However, it is not currently known if the c.882-40 T > A allele is a pathogenic mutation.

Eight patients with available genetic information had the p.Pro1007Ala mutation. Cases were fairly evenly distributed between the late-infantile, juvenile and adolescent/adult-onset subgroups.

Full genome sequencing performed in a number of patients with one identified mutation failed to detect any second mutation. These patients (and their listed genotypes) were: 11 (c.2819C > T(p.Ser940Leu/?), 18 (c.2008_2011del(p.Cys670ProfsX17/?), 28 (c.3182 T > C(p.Ile1061 Thr)/?), 31 (c.3182 T > C(p.Ile1061Thr)/?), 51 (c.3467A > G(p.Asn1156Ser)/?), 52 (c.3467A > G(p.Asn1156Ser)/?), 53 (c.3591 + 4delA/?) and 66 (c.1211G > A(p.Arg404Gln)/?).

### Miglustat use

The information regarding miglustat use should be regarded in recognition of the year during which miglustat became commercially available in the EU (2009), and of the time of data cut-off. Overall, 51 patients (35 %) received miglustat during the observation period (Tables 2–6): 2/8 (25 %) early infantile-onset patients and 17/51 (33 %), 20/42 (48 %) and 12/25 (48 %) patients in the late infantile-, juvenile-, and adolescent/adult-onset subgroups, respectively. No patients with the neonatal form of NP-C and no patients in the ‘non-neurological’ subgroup received miglustat.

The mean ages at treatment start ranged from 0.5 to 49.0 years across the age-at-onset subgroups. Among a total of 38 patients with evaluable information on treatment duration, the overall estimated mean (SD) treatment duration was 2.6 (2.3) years: median (range) 2.0 years (1 week to 8 years). Approximate mean (SD) durations per evaluable age-at-onset subgroup were: 2.6 (2.0) years in late infantile-, 2.8 (2.4) years in the juvenile-, and 2.9 (2.6) years in the adolescent/adult-onset subgroups.

### Disease course among related patients

A total of 44 patients from 20 families in this cohort were siblings, most of whom (38 [86 %]) had at least one identified *NPC1* mutant allele (see Additional file 1: Table S1). No NP-C gene mutations were identified in three sibling pairs: diagnoses were established by other means based on clinical symptoms and/or laboratory biochemical (filipin testing) and histological methods.

In general, ages at neurological onset and clinical phenotypes appeared congruent between siblings with identical mutant genotypes. Exceptions included patient 6, the sister of patients 25 and 26, all of whom were homozygous for the c.3020C > T (p.Pro1007Leu) mutant allele. Patient 6 had the neonatal-onset form of NP-C, neonatal liver disease and hepatosplenomegaly, and died aged 2 months due to failure to thrive and severe chest infection. Her sisters are twins and have late-infantile onset NP-C. Both twins also had neonatal jaundice and hepatosplenomegaly, with a variety of characteristic neurological signs observed from before the age of 4 years, but both were still alive at the time of data cut-off.

Another sibling pair (patients 80 and 108) were both heterozygotes with c.3182 T > C (p.Ile1061Thr) and c.3019C > G (p.Pro1007Ala) mutant alleles. The older sibling (patient 108) has adolescent/adult-onset NP-C, presented with VSGP, developmental delay and psychiatric disturbances in his late-teens, and survives aged 39 years at last follow up: he had received miglustat therapy for 3 years at data cut-off. His brother (patient 80) had juvenile-onset NP-C and presented with developmental delay and VSGP before 10 years of age. He also had severe seizures that are considered likely to have contributed to his death ‘due to NP-C’ at the age of 18 years.

Finally, patients 139 and 140 both have no neurological manifestations. While patient 139 was diagnosed at an early age following investigations of neonatal liver disease and organomegaly, his older brother has no record of systemic manifestations, and was diagnosed (based on genetic testing) due to mainly to his sibling disease history.

## Discussion

It is important to continue gathering knowledge on the natural history of NP-C in order to aid in the clinical management and targeted therapy of affected patients, and to aid in providing counselling and support for their families and caregivers. This summary of data from 146 historical and current UK-based NP-C patients analysed between 1999 and end-2011 provides findings from the largest national NP-C cohort reported to date. Although some patients have minimal data available, they are still included in this report to indicate the full extent of the UK NP-C cohort and provide as full a clinical picture of diagnosed patients as possible.

Based on recent estimates of the total UK population (64.1 million) and national birth rate (12.27/1000) the current birth prevalence of NP-C in the UK is 0.78 cases per 100,000 births, which seems roughly in line with previous estimates of birth prevalence of NP-C in Western Europe (France, Germany and the UK) over the period 1988–2002 [21, 22]. Based on records of all known UK cases diagnosed since 1985, the rate of diagnoses per decade (i.e., the mean number across all years within each decade) has risen steadily over the last three decades, from 3.5 new cases/year during 1985–1994 to 5.1 new cases per year during 1995–2004, and 6.0 new cases per year during 2005–2011. In addition, a total of 21 new cases have been diagnosed between 2012 and 2015, although these most recent cases are not included in this cohort analysis due to non-availability of access to full clinical information after data cut-off. This trend possibly reflects increased awareness of the disease over the last two decades and, in particular, improvements in genetic analysis methods for confirmation of disease-causing mutations.

Data on visceral symptoms in this cohort were in line with baseline data from 163 patients included in the International NP-C Registry [19]. Neonatal liver disease and/or prolonged jaundice, or a history of just neonatal jaundice, was a feature in all age subgroups in this cohort. As could be expected, neonatal jaundice was recorded in many patients (50–100 %) in the infantile-onset subgroups, and in far lower proportions of patients (0–33 %) in the juvenile- and adolescent/adult-onset subgroups. There was a clear trend for decreased occurrence of organomegaly with increasing age at neurological onset, although organomegaly was not as common in the neonatal-onset subgroup as it was among early-infantile onset patients due to its incremental nature. Nevertheless, splenomegaly is generally considered a consistent indicator of possible NP-C in neonates, and has often served as a signal leading to early diagnosis.

NP-C has been reported as the second most common genetic cause of liver disease during infancy in the UK, after alpha-1-antitrypsin deficiency [23, 24], and investigations to exclude NP-C are common practice in liver disease/neonatal units. A large proportion of early-onset patients in this cohort underwent extensive liver investigations during the neonatal period, but in many cases NP-C was subsequently diagnosed years later, after the appearance of neurological signs. This seems typical of the NP-C population as a whole, as reflected by substantial delays to diagnosis in many cases, globally, and reflects the need for clinicians in all specialities to take a full clinical history when seeing new patients.

Based on previous clinical observations, neonatal jaundice without other overt signs of liver disease can herald a more aggressive clinical course of NP-C, particularly if neurological abnormalities appear during the first 4 years of life [15, 24]. However, the degree of neonatal liver disease does not appear to be a reliable indicator of future disease progression, as illustrated by cases where neurological manifestations became apparent during adulthood despite a history of neonatal liver disease. It is important to consider that early visceral symptoms lack prognostic value in NP-C during family counselling for NP-C cases diagnosed during the neonatal period, providing advice for planning ahead for the emotional and financial burdens of the disease [25]. Of particular note are the sibling patients 132 and 141, who share identical novel heterozygous *NPC1* mutations. Both had very severe neonatal liver disease necessitating intensive care, and were followed up over a course of years by their hepatologist. Patient 141 had non-neurological NP-C and died at the age of 36 due to a severe infection in late pregnancy: post-mortem examination showed no storage or neurological problems. Her brother (patient 132) was still alive and self-caring at data cut off, and displayed mild cognitive impairment at last follow up.

The profile of neurological manifestations recorded in this cohort was consistent with other large-cohort NP-C studies [2, 4, 16, 19, 26], with over half of patients displaying one or more of: ataxia; VSGP; dysarthria; dysphagia; and seizures/cataplexy. In terms of age at neurological onset, two-thirds of patients fell into the late infantile-onset and juvenile-onset subgroups. Given the frequency of childhood-onset forms of NP-C in this UK cohort up to the end of 2011, it is not surprising that developmental delay or cognitive deterioration were the most frequently recorded neurological manifestations. Similarly, the frequency of seizures and/or cataplexy was reflective of the overall young age at onset among UK patients, as seizure activity has generally been reported most frequently among patients with late-infantile and juvenile-onset disease [1].

It is notable that ataxia, which is a central component of the recently developed NP-C suspicion index (SI) for the detection of NP-C among patients with suggestive clinical symptoms [9], was the second-most common neurological manifestation in this cohort. VSGP is recognised as one of the earliest signs of neurological deterioration in NP-C [1, 9, 27, 28], so it is unsurprising that this neurological sign was also common. Excluding neonatal cases, VSGP was observed in over half of all UK patients, and was most frequent in those with the juvenile-onset (classical) form of NP-C.

Psychiatric manifestations were only recorded in patients in the juvenile and adolescent/adult-onset subgroups, which is in agreement with previous screening data [29] and numerous other published findings [1, 2, 30–34]. While little information was available on the precise types of psychiatric disorders for this analysis, patients with juvenile-onset NP-C tend to manifest behavioural problems, impaired learning, and expressive language disorder, which often culminate in failure at school [1]. Patients with adolescent/adult-onset NP-C and psychiatric disorders often exhibit schizophrenia-like psychosis (in up to 25 % of cases), but can also be diagnosed with bipolar disorder, depression and obsessive-compulsive disorder [1, 34].

While the severe neonatal and early-infantile onset forms of NP-C resulted in rapid deterioration and early mortality in the current cohort, total mortality decreased with increasing age at neurological onset among the late-infantile (59 %), juvenile (45 %) and adolescent/adult-onset (32 %) subgroups. Mean age at death values reflected this trend. Some adolescent/adult-onset patients have survived into their fifth and sixth decade of life, which confirms the more insidious, mild course of neurological deterioration that has been reported before among adults with NP-C [1, 26]. Indeed, a recent case has been reported of a 66 year-old female who remains free of any neurological or psychiatric manifestations 18 years after initial presentation [35].

Wide genetic variability coupled with a high degree of phenotypic heterogeneity make genotype–phenotype correlations difficult in NP-C. Variable clinical phenotypes have even been observed in monozygotic twins with the same genetic mutation [36]. Overall, 53/194 (27 %) mutations identified in this cohort were classified as novel. The exon 21 (p.I1061Thr) mutant allele of *NPC1* has previously been associated with the common juvenile-onset form of the disease, and is associated with a relatively well characterised cellular function and biochemical phenotype [37–39]. The overall prevalence of this allele has been quoted as approximately 15 % [21], although more recently it has been reported as particularly frequent (in 20–25 % of diagnosed cases) in France and the UK [2]. Based on this cohort update, the p.I1061Thr mutant allele was present in 38 % of patients overall, occurring in approximately equal proportions of patients in the late-infantile and juvenile-onset subgroups. The incidence of the second most frequent known *NPC1* mutation – p.1007Ala – appeared relatively much lower, occurring in the heterozygous state in only eight patients overall. The prevalence range for p.1007Ala alleles among the late-infantile, juvenile and adolescent/adult-onset subgroups was 4–10 %.

Interestingly, the prevalence of p.I1061Thr mutations was particularly high in the non-neurological subgroup, where it occurred in 50 % of patients. This begs the question of whether some patients currently in the non-neurological group might progress to develop neurological manifestations in the future. For instance, ‘non-neurological’ patient 136 had no neurological symptoms at last documented follow up aged 2.5 years. However, other patients with homozygous p.I1061Thr mutations (patients 46 and 81–84) all had late-infantile or juvenile onset neurological symptoms. In the same way, many patients with heterozygous p.I1061Thr mutations developed neurological symptoms during the late-infantile period (up to 6 years of age, *n* = 21), the juvenile period (*n* = 14), or during adolescence/adulthood (*n* = 7). Further follow-up might therefore result in re-categorisation of some or all of the ‘non-neurological’ p.I1061Thr patients as having symptomatic neurological disease. Nevertheless, the same cannot be said for eight patients in the non-neurological group in whom p.I1061Thr mutations have been excluded, and/or in whom no known *NPC1* mutant alleles have been detected.

Substrate reduction therapy (SRT) using miglustat has been shown to be effective in treating adult patients with Gaucher disease [40–44], and subsequently in the treatment of progressive neurological manifestations in children and adults with NP-C [1, 28, 45–48]. In total 34 % of UK patients have received miglustat therapy for any period of time. Longitudinal analyses of serial clinical status assessments are required to determine the impact of miglustat on neurological disease progression in this cohort.

## Conclusions

In summary, the prevalence of NP-C in the UK is in line with previous observational data from other European cohorts, and it is notable that the annual rate of diagnosis of the condition has increased over the past 30 years, possibly due to increasing awareness and improvements in molecular diagnostic methods. The wide phenotypic variability and the overall profile of genotypes detected among UK patients are also consistent with data from other Western populations. In particular, age at onset of neurological manifestations once more appeared associated with more rapid disease progression and a lower age at death. Further analyses are required to assess the impact of miglustat therapy on neurological disease progression.

## Additional file

Additional file 1: Table S1. Comparison of sibling profiles. (DOCX 42 kb)

## Abbreviations

LDL: low-density lipoprotein
NP-C: Niemann-Pick disease type C
*NPC1/NPC2*: genes affected in NP-C
SD: standard deviation
SRT: substrate reduction therapy
VSGP: vertical supranuclear gaze palsy
