# Supplementary material for: Observational cohort study of the natural history of Niemann-Pick disease type C in the UK: a 5-year update from the UK clinical database
Source: BMC Neurol. 2015 Dec 15;15:257. doi: 10.1186/s12883-015-0511-1 (PMC4678528; doi:10.1186/s12883-015-0511-1)
Supplement: Additional file 1: Table S1. — Comparison of sibling profiles. (DOCX 42 kb) [file 12883_2015_511_MOESM1_ESM.docx]

**Supplementary Table 1.** Comparison of sibling profiles

| Patient number/ Gender | Sibship | Age subgroup | Age at diagnosis | Age at last FU (y, m) /death (†) | Neonatal LD | HS/S | Seizures /cataplexy | VSGP | Dev. delay/ cognitive problems | Ataxia | Swallowing problems | Psychiatric disturbance | Slurred speech | Miglustat? | Age at 1^st^ miglustat start | Miglustat duration | Genetic mutations | |
| --- | --- | --- | --- | --- | --- | --- | --- | --- | --- | --- | --- | --- | --- | --- | --- | --- | --- | --- |
| 2/F | a | Neo | PM | 0^†^ | FA | – | – | – | – | – | – | – | – | No | – | – | – | |
| 3/F | a | Neo | PM | 0^†^ | FA | – | – | – | – | – | – | – | – | No | – | – | – | |
| 4/M | b | Neo | Birth | 1m^†^ | Yes | Yes | – | – | – | – | – | – | – | No | – | – | c.3501C>G(p.Phe1167Leu)/c.3501C>G(p.Phe1167Leu) | |
| 5/M | b | Neo | Birth | 2m^†^ | Yes | Yes | – | – | – | – | – | – | – | No | – | – | c.3501C>G(p.Phe1167Leu)/c.3501C>G(p.Phe1167Leu) | |
| 60/M | b | LI | 8y | 16y^†^ | PJ | No | E 16y | Yes | 8y | 5y | 12y | – | Yes | No | – | – | c.3501C>G(p.Phe1167Leu)/c.3501C>G(p.Phe1167Leu) | |
| 92/F | b | JUV | 3m | 17y 2m^†^ | PJ | No | E 15y | 11y | 10y | 10y 5m | 15y | No | Yes | Yes | – | – | c.3501C>G(p.Phe1167Leu)/c.3501C>G(p.Phe1167Leu) | |
| 6/F | c | Neo | 2m | 7m^†^ | Yes | Yes | – | – | – | – | – | – | – | No | – | – | c.3020C>T(p.Pro1007Leu)/c.3020C>T(p.Pro1007Leu) | |
| 25/F | c/twin | LI | At birth | 5y 6m | PJ LD | HS | No | 4y | <4y | <4y | No | – | 4y | Yes | 4y | 1y | c.3020C>T(p.Pro1007Leu)/c.3020C>T(p.Pro1007Leu) | |
| 26/F | c/twin | LI | At birth | 5y 6m | PJ LD | HS | No | 4y | <4y | <4y | No | – | 4y | Yes | 4y | 1y | c.3020C>T(p.Pro1007Leu)/c.3020C>T(p.Pro1007Leu) | |
| 11/M | d | EI | PM | 7y 8m^†^ | No | No | C 2y E 4y | Yes | <2y | <2y | 5y | – | Yes | No | – | – | c.2819C>T(p.Ser940Leu/? | |
| 23/F | d | LI | 3y 9m | 12y 9m^†^ | No | No | C 6y | 6y | 6y 5m | 4y 7m | 7y | – | Yes | Yes | – | – | c.2819C>T(P.Ser940Leu)/? | |
| 12/F | e twin | EI | 11m | 8y 5m^†^ | PJ | Yes | C 5y E 5y 10 m | 3y | Yes | Never mobile | No | – | <3y | Yes | 7y | 1w | c.3107C>T(p.Thr1036Met/c.3557G>A(p.Arg 1186His) | |
| 13/F | e twin | EI | 11m | 7y 1m^†^ | PJ | Yes | C 5y E 5y 10 m | 3y | Yes | Never mobile | No | – | <3y | Yes | 7y | 1w | c.3107C>T(p.Thr1036Met/c.3557G>A(p.Arg 1186His) | |
| 15/M | f | LI | 2y | 5 y 4m^†^ | – | <1y 8m | E <4y 11m | – | <4y 6m | <4y 6m | <4y 6m | – | <3y | No | – | – | c.3020C>T(p.Pro1007Leu)/c.3020C>T(p.Pro1007Leu) | |
| 27/F | f | LI | At birth | 3y | Yes | Yes | No | No | <3y | Mild | No | – | No | Yes | 0.5y | 2.5y | c.3020C>T(p.Pro1007Leu)/c.3020C>T(p.Pro1007Leu) | |
| 21/M | g | LI | 2y 11m | 7y^†^ | PJ | HS | C 4y | 2y 11m | 3y | <4y | Yes | – | <4y | No | – | – | c.2464_2465insT(p.Lys822IlefsX48)/c.2201G>T(p.ser734Ile) | |
| 22/F | g | LI | 4y | 6y 10m | Slight jaundice | No | No | <6y | <5y | <6y | No | – | <6y | Yes | 3y | 3y | c.2464_2465insT(p.Lys822IlefsX48)/c.2201G>T(p.ser734Ile) | |
| 32//M | h | LI | 2y | 11y 2m^†^ | PJ LD | No | E 6y 5m | Yes | 4y | Yes | Yes | – | – | No | – | – | c.3182T>C(p.Ile1061Thr)/? | |
| 33/M | h | LI | 1y | 8y 5m^†^ | PJ LD | No | C 6y E 6y | No | <5y | – | 8y | – | – | No | – | – | c.3182T>C(p.Ile1061Thr)/? | |
| 51/F | i | LI | 8y 6m | 27y 5m | No | 2y | C 5y | 8y | 13y | 5y | 13y | – | 11y | No | – | – | c.3467A>G(p.Asn1156Ser)/? | |
| 52/F | i | LI | 14y 5m | 33y 5m^†^ | No | No | E 13y | 14y | 5y | 13y | 13y | – | 12y | No | – | – | c.3467A>G(p.Asn1156Ser)/? | |
| 53/F | j | LI | 8y 4m | 16y 11m | No | No | E 7y 5m C 8y | 6y 9m | 3y 5m | 4y | 8y 6m | – | Yes | No | – | – | c.3591+4delA/? | |
| 93/F | j | JUV | 10y | 13y 6m | No | No | Yes | Yes | Yes | Yes | Yes | No | Yes | Yes | 10y | 3y | c.3591+4delAla/? | |
| 55/M | k | LI | – | 10y 5m^†^ | No | No | C 6y | 5y | 5y | 5y | 5y | – | Yes | No | – | – | – | |
| 63/M | k | LI | – | – | – | – | – | Yes | Yes | Yes | Yes | – | Yes | No | – | – | – | |
| 57/F | l | LI | 2y | 6y^†^ | No | HS 6m | No | 3y | 2y 5m | 2y 5m | 5y | – | No | No | – | – | – | |
| 61/M | l | LI | 7y 1m | 17y 5m^†^ | No | S 2y 6m | C 10y | 7y 1m | 4y 5m | 5y 5m | 10y | – | Yes | No | – | – | – | |
| 80/M | m | JUV | 12y | 18y 8m^†^ | No | S 2–10y | E 11y | 10y | 8y | 10y | 18y | No | Yes | No | – | – | c.3182T>C(p.Ile1061Thr)/c.3019C>G(p.Pro1007Ala | |
| 108/M | m | AA | 23y | 35y 8m | PJ | No | No | 17y | 17y | 25y | 25y | No | Yes | Yes | 32y | 3y | c.3182T>C(p.Ile1061Thr)/c.3019C>G(p.Pro1007Ala) |  |
| 102/F | n | JUV | Teens | 23y^†^ | No | No | – | No | Teens | Teens | Teens | 23y | – | No | – | – | c.3182T>C(p.Ile1061Thr)/? |  |
| 112/M | n | AA | 24y | 40y 2m | No | No | No | 16y | 24y | <24y | No | 29y | 32y | No | – | – | c.3182T>C(p.Ile1061Thr)/? |  |
| 106/F | o | JUV | 27y | 35y 10m | No | No | No | 27y | 13y | 26y | 26y | No | 27y | Yes | – | – | c.1552C>T(p.Arg518Trp)/c.1552C>T(p.Arg518Trp) |  |
| 107/M | o | JUV | 30y | 37y 2m^†^ | No | No | Teens | 26y | 26y | 26y | 27y | 25y | 26y | No | – | – | c.1552C>T(p.Arg518Trp)/c.1552C>T(p.Arg518Trp) |  |
| 117/F | o | AA | 25y | 33y 11m | No | No | No | No | 25y | 25y | 32y | No | 32y | Yes | 29y | 4y | c.1552C>T(p.Arg518Trp)/c.1552C>T(p.Arg518Trp) |  |
| 113/M | p | AA | 19y | 43y 2m | No | S 18y | E 17y | 25y | 18y | 18y | 25y | 17y | 18y | No | – | – | c.1843C>T(p.Arg615Cys)/c.3289-3291del(p.Asp1097del) |  |
| 115/M | p | AA | 18y | 28y 8m^†^ | No | S 1y 4m | No | Yes | Yes | Yes | 25y | 28y | Yes | No | – | – | c.1843C>T(p.Arg615Cys)/c.3289-3291del(p.Asp1097del) |  |
| 123/M | q | AA | 40y | 44y 5m | – | No | Tremor | <40y | <40y | <40y | No | No | No | Yes | 43y | 1y | c.1408G>C(p .Ala 470 Pro/c.1816G>C (p.Glu608Gln) |  |
| 130/M | q | AA | 40y | 41y 9m | – | 40y | Tremor (35y) | Yes | No | Yes | 40y | No | No | Yes | 39y | – | c.1408G>C(p .Ala 470 Pro/c.1816G>C (p.Glu608Gln) |  |
| 125/F | r | AA | 29y | 30y 3m | No | 20y | No | 20s | 20s | 20s | 20s | Teens | 20s | – | – | – | c.2764C>T(p.Gln922X)/c.1133T>C(p.Val378Ala) |  |
| 126/M | r | AA | 25y | 26y 8m | No | No | No | 25y | Mild 25y | No | No | No | No | Yes | 25y | 1y | c.2764C>T(p.Gln922X)/c.1133T>C(p.Val378Ala) |  |
| 132/M | s | 1974 | Early 30s | – | – | – | – | – | Yes | – | – | – | – | Yes | – | – | c.2336del (p.Phe779SerfsX2)/c.2621A>T (p.Asp874Val) |  |
| 141/F | s | 1971 | 1y5m | 36y 8m^†^ | PJ LD | Birth | No | No | – | No | No | No | No | No | – | – | c.2336del (p.Phe779SerfsX2)/c.2621A>T (p.Asp874Val) |  |
| 139/M | t | 2010 | <1y | 1y | Yes | Yes | No | No | No | No | No | No | No | No | – | – | c.3182T>C (p.Ile1061Thr)/c.3289G >A (p.Asp1097Asn) |  |
| 140/M | t | 2006 | – | 5y | No | No | No | No | No | No | No | No | No | No | – | – | c.3182T>C (p.Ile1061Thr)/c.3289G>A (p.Asp1097Asn) |  |

*AA, adolescent/adult-onset NP-C; '–', no data/not known; C, cataplexy; E, epilepsy; EI, early-infantile onset NP-C; FA, foetal ascites; HM, hepatomegaly; HS/S, hepatosplenomegaly/splenomegaly; LD, liver disease; LI, late-infantile onset NP-C; LTx, liver transplant; m, months; NC, neonatal cholestasis; Neo, neonatal onset NP-C; PJ, prolonged jaundice; PM, post mortem; y, years; w, weeks.*
